# Understanding insulin resistance pathophysiology in PCOS: a genetic approach

**DOI:** 10.1186/1755-8166-7-S1-P92

**Published:** 2014-01-21

**Authors:** Srabani Mukherjee, Nuzhat Shaikh, Roshan Dadachanji, Nalini Shah, Anushree Patil

**Affiliations:** 1 National Institute for Research in Reproductive Health, J.M. Street, Parel, Mumbai- 400012, India; 2 Department of Endocrinology, Seth GS Medical College, Mumbai- 400012, India

## 

Polycystic ovary syndrome (PCOS) is a common endocrine disorder which affects women in their childbearing years, characterized by chronic anovulation, hyperandrogenemia, hyperinsulinemia and obesity. It is frequently associated with an increased risk for insulin resistance with long term health implications like type 2 diabetes mellitus and atherosclerosis disease. However, the aetiopathogenesis of this syndrome still remains elusive. The familial clustering of women with PCOS suggests that heredity is implicated in the origin of this syndrome. PCOS is now considered a polygenic trait that might result from the interaction of various susceptible and protective genomic variants along with the influence of environmental factors. A candidate gene based approach for investigating association of genes involved in insulin resistance such as INSR, PPARγ, and PON1 with PCOS and its related phenotypes in Indian women has been undertaken. Clinical and biochemical parameters to evaluate PCOS associated traits related to metabolic and hyperandrogenemia were also assessed in all the participants. A polymorphism in insulin receptor gene (INSR), C/T His1058 showed association with PCOS only in lean women and also with hyperandrogenemia and hyperinsulinemia traits. Peroxisome proliferator activated receptor gamma (PPARγ) is a transcription factor that regulates glucose, lipid homeostasis and intracellular insulin signaling. The most common variant Pro12Ala of PPARγ which has a role in preserving insulin sensitivity showed significant association with decreased PCOS susceptibility while the other His447His polymorphism along with former was significantly associated with lower insulin related traits in women with PCOS. Paraoxonase 1 (PON1) gene encodes an antioxidant enzyme which protects against LDL oxidation and thus prevents atherosclerosis. Serum paraoxonase levels were significantly reduced in our PCOS group. A coding region L55M SNP showed association with PCOS. The study establishes that in Indian women, variations in genes related to insulin resistance do have an influence on the pathophysiology of PCOS.

